# Nitric oxide and phytohormone interactions: current status and perspectives

**DOI:** 10.3389/fpls.2013.00398

**Published:** 2013-10-09

**Authors:** Luciano Freschi

**Affiliations:** Laboratory of Plant Physiology and Biochemistry, Department of Botany, University of Sao PauloSao Paulo, Brazil

**Keywords:** nitric oxide, plant hormones, auxin, cytokinin, gibberellin, abscisic acid, ethylene, *S*-nitrosylation

## Abstract

Nitric oxide (NO) is currently considered a ubiquitous signal in plant systems, playing significant roles in a wide range of responses to environmental and endogenous cues. During the signaling events leading to these plant responses, NO frequently interacts with plant hormones and other endogenous molecules, at times originating remarkably complex signaling cascades. Accumulating evidence indicates that virtually all major classes of plant hormones may influence, at least to some degree, the endogenous levels of NO. In addition, studies conducted during the induction of diverse plant responses have demonstrated that NO may also affect biosynthesis, catabolism/conjugation, transport, perception, and/or transduction of different phytohormones, such as auxins, gibberellins, cytokinins, abscisic acid, ethylene, salicylic acid, jasmonates, and brassinosteroids. Although still not completely elucidated, the mechanisms underlying the interaction between NO and plant hormones have recently been investigated in a number of species and plant responses. This review specifically focuses on the current knowledge of the mechanisms implicated in NO–phytohormone interactions during the regulation of developmental and metabolic plant events. The modifications triggered by NO on the transcription of genes encoding biosynthetic/degradative enzymes as well as proteins involved in the transport and signal transduction of distinct plant hormones will be contextualized during the control of developmental, metabolic, and defense responses in plants. Moreover, the direct post-translational modification of phytohormone biosynthetic enzymes and receptors through *S*-nitrosylation will also be discussed as a key mechanism for regulating plant physiological responses. Finally, some future perspectives toward a more complete understanding of NO–phytohormone interactions will also be presented and discussed.

## INTRODUCTION

As sessile organisms, plants must rely on highly sophisticated signaling mechanisms to adjust their growth, shape, and metabolism with the constant changes in their environment. Playing a key role in this process, plant hormones integrate a multitude of internal and external cues into coordinated metabolic and developmental responses, which, in turn, maximize plant fitness under diverse ontogenetic and environmental contexts. To effectively carry out such critical function, distinct plant hormones intensively interact among themselves and also with other endogenous signaling substances (Santner et al., 2009).

Among these hormone-interacting molecules, the gaseous free radical nitric oxide (NO) has recently gained special interest in the research community given its involvement in a number of signaling cascades controlling plant responses ranging from seed germination to plant senescence (Neill et al., 2003; Wilson et al., 2008; Mur et al., 2012a). Whereas great strides have been made in recent years in understanding the mechanistic relationship between NO and phytohormones in certain physiological responses (Leon and Lozano-Juste, 2011; Terrile et al., 2012; Feng et al., 2013), the exact nature of the interaction between these substances in many developmental, metabolic, and defense events still remains remarkably elusive. In some cases, for instance, it is known that both NO and plant hormones are able to influence a given response, but it is not clear whether they share a common signaling cascade or just modulate the same plant event via parallel, independent signaling pathways.

## NO SIGNALING MECHANISMS: WHERE DO WE STAND?

**FIGURE 1:**
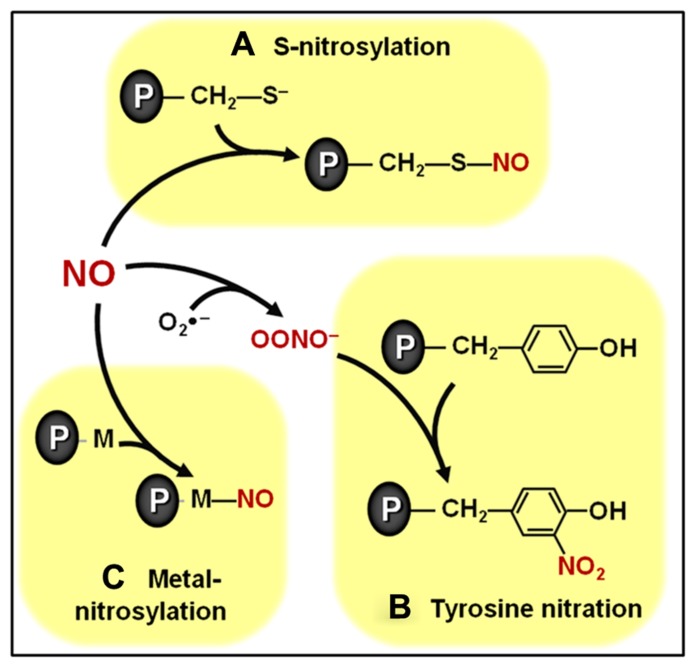
**Overview of biologically relevant NO-dependent post-translational modifications (PTMs). (A)** *S*-nitrosylation of cysteine residues. **(B)** Tyrosine nitration. **(C)** Metal nitrosylation. Proteins are represented with gray ovals and “P” letters.

As mentioned by Hancock et al. (2011), characterizing the precise function of NO in a particular signaling event is more difficult than it might appear. Firstly, the particular chemical characteristics of NO inexorably imply peculiar mechanisms for “sensing” the presence and levels of this signaling molecule. Instead of a unique or very few receptors, NO likely interacts with a wide range of target proteins via direct modification of protein structure (**Figure 1**). Through these chemical modifications of target proteins, NO may trigger changes in their activities and cellular functions, ultimately leading to the transduction of the NO message into plant responses.

Among the biologically relevant NO-dependent post-translational modifications (PTMs), the covalent modification of cysteine residues through a processes known as *S*-nitrosylation (**Figure 1A**) has been emerging as a critically important mechanism intermediating NO signal transduction in plants (Lindermayr et al., 2005; Astier et al., 2011, 2012). This specific, reversible and regulated NO-dependent PTM has been implicated as potentially controlling the function of components of plant processes as diverse as cellular architecture, photosynthesis, genetic information processing, protection against oxidative stress, defense responses to biotic and abiotic stresses, hormonal signaling, among others (Lindermayr et al., 2005; Romero-Puertas et al., 2008; Astier et al., 2011, 2012; Astier and Lindermayr, 2012). Currently, some of the best characterized examples of *S*-nitrosylation in plant systems include the modulation of phytohormone biosynthetic enzymes (Lindermayr et al., 2006), receptors (Terrile et al., 2012), and signal transduction proteins (Feng et al., 2013), which will be discussed in more detail later in this review. The specificity of this NO-triggered PTM is essentially based on the fact that only cysteine residues surrounded by particular neighboring amino acids seem to be the target of *S*-nitrosylation (Astier et al., 2011; Kovacs and Lindermayr, 2013).

A second physiologically relevant NO-dependent PTM depends on the reaction between NO and reactive oxygen species (ROS), such as superoxide ($O_{2}^{-}$), resulting in the production of NO-derived species, such as peroxynitrite (ONOO^-^), which, in turn, can covalently modify tyrosine residues through a process known as tyrosine nitration (**Figure 1B**; Astier and Lindermayr, 2012). Initially considered an irreversible process, tyrosine denitration is now believed to occur either enzymatically or non-enzymatically (Abello et al., 2009; Vandelle and Delledonne, 2011; Astier and Lindermayr, 2012). Reinforcing such reversibility in tyrosine nitration, transient, rather than permanent, changes in the abundance of nitrated proteins have already been reported in the literature (Cecconi et al., 2009). More research is required to better define the biological relevance of this NO-dependent protein modification in plants, which apparently may target proteins involved in many basic cellular processes, such as photosynthesis, respiration and nitrogen metabolism (Cecconi et al., 2009; Chaki et al., 2009b; Lozano-Juste et al., 2011; Tanou et al., 2012)

In addition to *S*-nitrosylation and tyrosine nitration, a third important NO-dependent PTM involves the binding of NO to transition metal centers of metalloproteins in a process known as metal nitrosylation (**Figure 1C**). Currently, one of the best characterized examples of metal nitrosylation is the activation of soluble guanylate cyclase (sGC) in animal systems (Ignarro et al., 1999). In plants, although cyclic guanosine monophosphate (cGMP) has already been reported as an important intermediate in several NO-induced processes, including root development, mitochondrial respiration, nodule functioning, and defense responses (Durner et al., 1998; Pagnussat et al., 2003; Ederli et al., 2008; Keyster et al., 2010; Wang et al., 2010), more studies are still required to clarify whether metal nitrosylation also regulates plant sGC.

**FIGURE 2:**
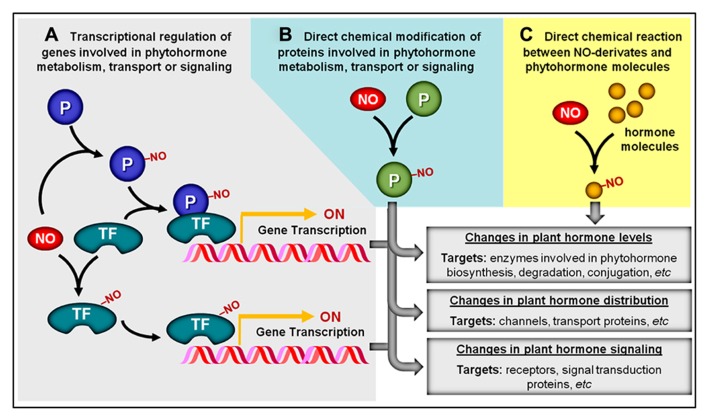
**Overview of potential NO–phytohormone interaction mechanisms. (A)** By chemically modifying transcription factors (TF) and other proteins (P), NO may influence the transcription level of genes involved in phytohormone metabolism, transport, or signal transduction. **(B)** NO may post-translationally modify proteins (P) directly involved in the production, distribution, or signaling of plant hormones. **(C)** NO or NO-derived reactive species might also chemically react with certain plant hormonal species, rendering products with altered biological activity. NO-dependent chemical modifications are represented by “–NO.”

Regardless of the specific type of NO-triggered PTM considered, these chemical modifications may represent a central mechanism through which NO impacts signaling networks responsible for controlling plant development and metabolism. In responses regulated by plant hormones, for instance, these PTMs might facilitate the influence of NO on hormonal production and/or action via three distinct but non-exclusive mechanisms. The first mechanism implicates NO-dependent chemical modifications of proteins (e.g., transcription factors, regulatory proteins, and channels) whose functions may not be directly implicated in plant hormone metabolism, distribution, or signaling but, instead, may influence the abundance of other proteins more intimately implicated in such specific roles (**Figure 2A**). In contrast, a second and more direct way involves the NO-triggered PTM of proteins directly associated with the production, degradation, conjugation, transport, perception, or signaling transduction of plant hormones (**Figure 2B**). For example, in the first mechanism, NO may chemically modify a transcription factor that stimulates the production of a hypothetical enzyme responsible for hormone degradation, whereas in the second mechanism, NO would directly interact and modify the activity, stability, and/or cellular localization of this degradative enzyme (**Figures 2A, B**). A third possibility recently described in the literature involves the direct chemical reaction between NO-derivates (e.g., peroxynitrite) and certain hormonal species (e.g., zeatin), rendering products with altered biological activity (**Figure 2C**). Specific examples of all three of these mechanisms of NO–phytohormone interaction will be provided and discussed later in this review.

## NO SIGNALING SPECIFICITY: HOW CAN SUCH A SMALL MOLECULE CONTROL SO MANY PROCESSES?

Considering that a massive number of proteins, peptides, and other molecules may undergo changes in their structure and activity via direct NO-dependent chemical modifications (Astier et al., 2011, 2012; Astier and Lindermayr, 2012) and an equivalent amount of genes may have their transcription levels influenced by NO (Polverari et al., 2003; Parani et al., 2004; Grun et al., 2006; Besson-Bard et al., 2009), one pertinent question that arises is how NO signals can confer sufficient specificity to trigger coordinated downstream effects. Although answering this question involves a certain degree of speculation at this point in the research of NO signaling in plants, aspects such as spatial and temporal signaling compartmentation and a precise control of NO biosynthesis and removal might possibly be key to explaining how a molecule as small as NO might be responsible for controlling so many plant responses.

As is the case with cytosolic Ca^2^^+^, a strict temporal and spatial regulation of NO levels inside each plant cell might be essential for delivering sufficiently specific NO signals. The transient generation of “NO hot-spots,” in particular plant cell compartments, could lead to compartmentalized protein modifications (Neill et al., 2008b), and, consequently, the NO signals may be sensed by a specific group of proteins responsible for a particular set of cellular functions. A possible mechanism for assuring such localized action of NO could be the existence of macromolecular modules including all major NO signaling components (e.g., NO biosynthetic enzymes, NO removal enzymes, and targets of NO-dependent PTMs). Although such macromolecular complexes have not yet been described in plants, recent models for NO-mediated stress signaling in animal systems suggest, for instance, that the control of certain membrane calcium channels via reversible *S*-nitrosylation is facilitated by the close proximity of these channels to the NO-generating enzyme (Stamler and Meissner, 2001). Therefore, in this case, instead of a global change in cellular NO levels, the transient production of this signaling molecule at particular regions of the animal cell may control the activity of nearby target proteins via reversible *S*-nitrosylation (Martinez-Ruiz et al., 2013). As an ultimate consequence, such compartmentation and fine-tuned dynamics of NO production could minimize a certain spatial promiscuity in terms of concomitant occurrence of NO, NO-derivates, and their target proteins.

A relevant bottleneck for advances in the evaluation of the possible existence of such NO signaling macromolecular modules in plants is the still incipient characterization not only of the targets of NO-dependent PTMs but also of the biosynthetic and removal machinery responsible for controlling NO levels inside the plant cell compartments. Interestingly, though, compartmentalized production of NO has already been reported in plant cells. Foissner et al. (2000), for example, reported that after challenging epidermal tobacco cells with the elicitor cryptogein, NO accumulation first appeared in the plastids and subsequently in other cell compartments, such as the nucleus and the cytoplasm.

## NO PRODUCTION AND REMOVAL: WHY SO MANY PATHWAYS IN PLANTS?

Placing NO as an element of a given signaling cascade necessarily implies that changes in its levels or cellular localization might occur during the course of the signaling event. Therefore, characterizing the specific changes in the NO biosynthetic and degradation mechanisms responsible for delivering adequate concentrations of this molecule at the right time and place seems a logical step in any research interested in discriminating the actual role of NO during the regulation of specific plant responses. However, the relevance of the different origins of NO in plants is still poorly understood; as a consequence, controversy and ambiguity are still frequently found in the current literature (Kaiser and Planchet, 2006; Gupta et al., 2011).

**FIGURE 3:**
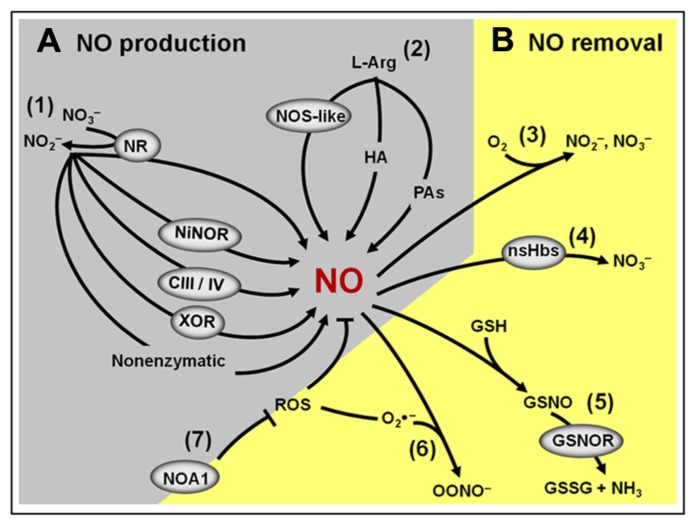
**Overview of the NO production and removal mechanisms in plants. (A)** Main components of the NO biosynthetic machinery: (1) Nitrite-dependent NO production in plants includes a non-enzymatic pathway and several enzymatic pathways involving the action of cytosolic and plasma membrane nitrate reductases (NR), nitrite–NO reductase (NiNOR), mitochondrial electron transport chain (CIII/IV) and xanthine oxidoreductase (XOR). (2) L-Arginine-dependent NO production pathway involves a non-identified nitric oxide synthase (NOS)-like enzyme and two still poorly characterized pathways using hydroxylamine (HA) or polyamines (PAs) as substrates. **(B)** Main components of the NO removal machinery: (3) the reaction of NO with molecular oxygen leads to the spontaneous production of nitrite and nitrate. (4) NO can react with non-symbiotic hemoglobins (nsHbs) resulting in nitrate formation. (5) Alternatively, NO may react with reduced glutathione (GSH) to form *S*-nitrosoglutathione (GSNO), which, in turn, can be converted into oxidized GSSG and ammonia by the action of GSNO reductase (GSNOR). (6) NO can also react with superoxide ($O_{2}^{-}$), resulting in the formation of peroxynitrite (OONO^-^). (7) By influencing the production of reactive oxygen species (ROS), NO-ASSOCIATED 1 (NOA1) protein indirectly impacts NO levels in plants.

Besides the non-enzymatic NO production, which is believed to occur only under very specific conditions (Bethke et al., 2004), so far, seven potential enzymatic sources of NO have been identified in plants (**Figure 3A**; Gupta et al., 2011). Among them, nitrate reductase (NR) and NO synthase-like (NOS-like) activities are currently considered as the most likely candidates for the production of NO under physiologically relevant conditions (Neill et al., 2008b; Mur et al., 2012a). Since the discovery that plant NR could produce NO both under *in vitro* and *in vivo* conditions (Harper, 1981), a great deal of evidence has indicated this enzyme as one of the major plant biosynthetic sources of NO (Rockel et al., 2002; Meyer et al., 2005; Kaiser et al., 2010). Supporting this view, pharmacological and genetic approaches in different plant species, organs, tissues, and experimental conditions have revealed that NR inhibition frequently results in decreased NO production (Planchet and Kaiser, 2006; Oliveira et al., 2009; Freschi et al., 2010; Kolbert et al., 2010; Lombardo and Lamattina, 2012). On the other hand, the existence of NOS-like activity in plants is exclusively supported by biochemical and pharmacological evidence since a canonical NOS gene or a mutant deficient in NOS-like-dependent NO production has not been identified in higher plants yet (Corpas et al., 2006; Gupta et al., 2011; Mur et al., 2012a). Thus far, the organism more closely related to higher plants in which such a gene was described is the photosynthetic microalgae *Ostreococcus tauri* (Foresi et al., 2010; Correa-Aragunde et al., 2013), which belongs to a basal branch of the flowering plant evolutionary tree.

In 2003, studies revealed that *NO Associated1* (*AtNOA1*), formerly described as *AtNOS1* (Guo et al., 2003; Guo and Crawford, 2005; Zemojtel et al., 2006), also significantly influences NO generation in *Arabidopsis*. However, according with the latest consensus in the literature, *AtNOA1* encodes a chloroplast-localized cGTPase probably involved in ribosome assembly and subsequent mRNA translation to proteins in this organelle (Flores-Perez et al., 2008; Moreau et al., 2008). Therefore, the reduced NO production observed in *noa1* mutants is currently interpreted as an indirect outcome of disturbances in chloroplast metabolism due to the lack of *AtNOA1 *function (Zemojtel et al., 2006; Gas et al., 2009). More recently, this mutant was crossed with the NR-deficient *nia1-nia2* mutant of *Arabidopsis*, generating a triple mutant (*nia1,2noa1,2*), which presented no detectable NO production and a range of physiological and developmental disturbances (Lozano-Juste and Leon, 2010a), thereby reinforcing the physiological importance of these pathways for determining the endogenous NO levels in plants.

Another important and frequently neglected aspect that may influence NO metabolism and signaling in plants is the presence of efficient mechanisms for removing the NO signal from a particular cell type or compartment as soon as it is no longer required. Besides the inherent chemical instability of NO in the presence of oxygen, this molecule might also be removed from plant tissues by several biochemical mechanisms (**Figure 3B**; Neill et al., 2008b; Mur et al., 2012a). Firstly, NO can be removed by reacting with ROS, such as superoxide anions, generating peroxynitrite. Secondly, NO may interact with plant proteins, such as non-symbiotic hemoglobins (nsHbs), which facilitates its oxidation to nitrate (Perazzolli et al., 2006). Finally, NO might also react with thiol proteins and peptides, resulting in the formation of *S*-nitrosothiols. In plant tissues, one of the most abundant low-molecular-mass *S*-nitrosothiols is the intracellular antioxidant glutathione, which may react with NO or with the NO-derivate N_2_O_3_, generating *S*-nitrosoglutathione (GSNO; Neill et al., 2008b; Mur et al., 2012a). The GSNO formed can spontaneously liberate NO or be metabolized by the enzyme *S*-nitrosoglutathione reductase (GSNOR), originating oxidized glutathione (GSSG) and NH_3 _(Barroso et al., 2006; Corpas et al., 2008b; Leterrier et al., 2011). Besides being an intracellular NO reservoir, GSNO may also be transported between cells, possibly playing a critical role as a vehicle of the NO signal throughout the plant body (Corpas et al., 2013).

## NO–PHYTOHORMONE INTERACTIONS: GENERAL MECHANISMS AND IMPLICATIONS

Before exploring the general mechanisms underlying the interactions between NO and phytohormones, it is worth mentioning that a great diversity of methodological approaches, experimental designs, and plant models have been used in NO research, which sometimes makes it difficult to directly compare the literature data. In terms of methodological approaches, for instance, a considerable variety of analytical techniques have been employed to determine NO levels in plant systems, including the Griess and the hemoglobin assays, electron spin resonance, laser-based photoacoustic detection, ozone-based chemiluminescence, and various fluorescent probes (reviewed by Vitecek et al., 2008 and Mur et al., 2011). As expected, these different methods provide distinct information. For example, it is always a challenge to compare results obtained by gas-phase NO detection techniques (e.g., chemiluminescence or laser photoacoustic) with fluorescent methods for *in situ* NO detection since these two groups of techniques differ greatly in their specificity, spatial resolution, and capacity to indicate the actual concentration of NO inside the target cells (Mur et al., 2011). Furthermore, evaluations of NO levels under the same experimental conditions by two or more independent methods, although recommended (Mur et al., 2012a; Gupta and Igamberdiev, 2013), are rarely carried out (Besson-Bard et al., 2008).

Besides measuring NO itself, alternatives to access NO and other RNS signaling inside the cells, such as the abundance of NO-triggered chemical modifications on proteins and peptides, have also recently drawn increasing attention of the plant research community, providing, in some cases, spectacularly relevant information. A number of technical options are currently available for such proposes, including the determination of *S*-nitrosothiol levels in plant extracts by reductive gas-phase chemiluminescence (Valderrama et al., 2007; Corpas et al., 2008b; Lee et al., 2008; Chaki et al., 2009a), immunolocalization of particular *S*-nitrosylated thiols or proteins (Barroso et al., 2006; Valderrama et al., 2007; Corpas et al., 2008a; Chaki et al., 2009a; Leterrier et al., 2011) or even proteomic profiling of proteins chemically modified by NO or NO-derivates (Lindermayr et al., 2005; Chaki et al., 2009b; Astier et al., 2011; Kovacs and Lindermayr, 2013), which, among other aspects, may facilitate the identification of the specific targets of NO-dependent PTMs in distinct plant responses.

Another relevant aspect to be considered in NO–phytohormone interaction studies is that the simple observation of changes in NO levels triggered by exogenous plant hormones does not necessarily imply a straightforward relationship between NO and the hormonal stimulus. Firstly, the exogenous application of a signaling substance might potentially induce global, unspecific changes in plant biochemistry, metabolism, and development. Secondly, modifications in NO levels might sometimes result from excessive levels of exogenous hormones; therefore, whenever possible, the actual concentration of particular phytohormone species inside the plant cells and tissues should be determined following the supplementation with these substances. Finally, some plant hormones may affect the biosynthesis and signaling of others (Santner et al., 2009); consequently, the establishment of a direct correlation between the pharmacological effect of a specific plant hormone on a given cellular response is not always an easy task. To overcome such a lack of specificity and potentially artificial effects, the use of transgenic and mutant plants with altered production, degradation or signaling of particular hormonal classes as well as a detailed characterization of several elements involved in phytohormone and NO metabolisms and signaling transduction have proven to be a powerful strategy for accessing the mechanistic relationship between these substances (Desikan et al., 2002; Leon and Lozano-Juste, 2011; Terrile et al., 2012).

Despite these methodological disparities and the limited literature information currently available, there is virtually no doubt that NO and phytohormones interact at multiple, diversified levels. Depending on the signaling cascade, NO has been demonstrated to act either upstream or downstream of plant hormones (Hancock et al., 2011; Simontacchi et al., 2013). Obviously, placing NO downstream of the hormonal stimuli in a signaling route necessarily means that the NO biosynthetic, degradation, conjugation, or deconjugation machinery may be affected at certain point between the perception of hormonal stimulus and the induction of the plant response. Therefore, the time period between the hormonal message input and the detection of changes in endogenous NO levels represents valuable information. In some cases, lag phases compatible with changes in the transcripts level or protein abundance of NO-synthesizing or removal enzymes have been reported (Pagnussat et al., 2002; Freschi et al., 2010). However, under some particular circumstances, the lag phase observed between the application of plant hormones and the rise in NO endogenous levels has been shown to be of just few minutes (Tun et al., 2001; Garcia-Mata and Lamattina, 2002; Huang et al., 2004; Tun et al., 2006; Sun et al., 2010), which indicates that the post-translational regulation of proteins involved in NO metabolism rather than their *de novo* synthesis might sometimes be implicated.

When acting upstream of phytohormones, NO seems able to modulate elements controlling either the plant hormone levels (e.g., biosynthetic, degradation, and conjugation enzymes), distribution (e.g., transport proteins) or signaling (e.g., receptors and signal transduction proteins). This modulation has been shown to occur either at the transcriptional (Bethke et al., 2007; Liu et al., 2009; Manjunatha et al., 2010; Xu et al., 2010; Leon and Lozano-Juste, 2011) or post-translational levels (Lindermayr et al., 2006; Terrile et al., 2012; Feng et al., 2013); however, some post-transcriptional or even translational regulation of hormone-related proteins by NO, although not yet demonstrated, cannot be ruled out.

Based on the basic information provided thus far, the current state-of-the-art of the interplay between NO and each one of the major classes of plant hormones [i.e., auxins, cytokinins, gibberellins (GAs), abscisic acid (ABA), and ethylene] will now be discussed. Although discussed here in an isolated manner, it is important to keep in mind that very frequently, if not always, plant hormones intensively interact with each other during the induction and establishment of plant responses. However, future studies will still be required to mechanistically explain exactly how distinct plant hormones concomitantly interact with NO to regulate specific plant events.

### NO AND AUXINS INTERACTIONS

Synergistic effects of auxin and NO have been observed during the regulation of a series of plant responses, including root organogenesis (Pagnussat et al., 2002, 2003, 2004; Lanteri et al., 2006), gravitropic responses (Hu et al., 2005), root nodule formation (Pii et al., 2007), root responses to iron deficiency (Chen et al., 2010), activation of cell division and embryogenic cell formation (Ötvös et al., 2005), NR activity stimulation (Du et al., 2008), among others. In virtually all of these cases, NO was identified to function downstream of auxins, apparently through linear signaling pathways. Increased NO production has frequently been observed after exogenous auxin application (Pagnussat et al., 2002; Correa-Aragunde et al., 2004; Hu et al., 2005; Lombardo et al., 2006) or in auxin overproducer mutants (Chen et al., 2010), being especially evident in plant tissues or cells undergoing auxin-dependent physiological responses. On the other hand, no or weak stimulation in NO production by auxins has been reported in some particular experimental conditions or cell types (Tun et al., 2001; Guo et al., 2003), suggesting that the auxin-dependent NO production may occur exclusively under specific temporal and spatial contexts (Hu et al., 2005).

Currently, most of the reports on NO and auxin interaction are focused on plant root responses, with relatively little information available on the crosstalk between these two signaling molecules in shoot or reproductive tissues. During the last decade, detailed information about the interaction between NO and auxin during root growth and development was provided by a series of studies conducted by Lamattina and colleagues, including the interplay between these molecules during adventitious roots formation (Pagnussat et al., 2002, 2003, 2004), lateral root development (Correa-Aragunde et al., 2004), and root hair initiation and elongation (Lombardo et al., 2006). In almost all of these studies, the removal of NO by scavengers significantly decreased typical auxin-dependent root responses, such as the activation of mitogen-activated protein kinases (MAPKs) during the adventitious root formation (Pagnussat et al., 2004) and induction of cell cycle genes during lateral root formation (Correa-Aragunde et al., 2006).

Also focusing on root tissues responses, Chen et al. (2010) identified a direct correlation between auxin availability, root NO levels and the expression of iron acquisition genes and other Fe deficiency-associated stress responses, providing further support for the action of NO as a downstream element in the auxin signaling pathway. Similarly, a clear spatial correlation was also observed between the asymmetric auxin distribution and the endogenous NO localization during the gravitropic bending in soybean roots (Hu et al., 2005) and during indeterminate nodule formation in roots of *Medicago* species infected by auxin-overproducing rhizobia (Pii et al., 2007).

A possible role for NR as the major biosynthetic source of the auxin-induced NO production during some plant root responses has been suggested (Kolbert and Erdei, 2008). Kolbert et al. (2008), for instance, reported that the NO production during the auxin-induced lateral root development in *Arabidopsis* requires NR activity since the NR-deficient double mutant *nia1,nia2* failed to increase NO generation in response to exogenous auxin, whereas no evidence for an involvement of NOS in this response was observed. NR-dependent NO production was also shown to be crucially important for the adequate vesicle trafficking during root hair formation because exogenous NO application completely restored the abnormal vesicle formation and trafficking as well as root hair growth in the *nia1,nia2 Arabidopsis *mutant (Lombardo and Lamattina, 2012). In a few cases, however, such as during the auxin-regulated NO generation under Fe deficiency and during the gravitropic bending in soybean roots, evidence indicates the involvement of not only NR but also NOS and/or NOA1 in the auxin-induced NO generation (Hu et al., 2005; Chen et al., 2010).

Considering that many of these root responses, including root hair formation and lateral root development, respond to both auxins and nitrate supply, NR-dependent NO generation might be a key integrator of exogenous and endogenous cues leading to the control of plant root biology. Although the precise mechanism through which auxin trigger NR-dependent NO generation has still not been fully characterized, literature data indicate a promotive effect of this plant hormone on NR protein, activity and gene transcription (Vuylsteker et al., 1997; Du et al., 2008).

**FIGURE 4:**
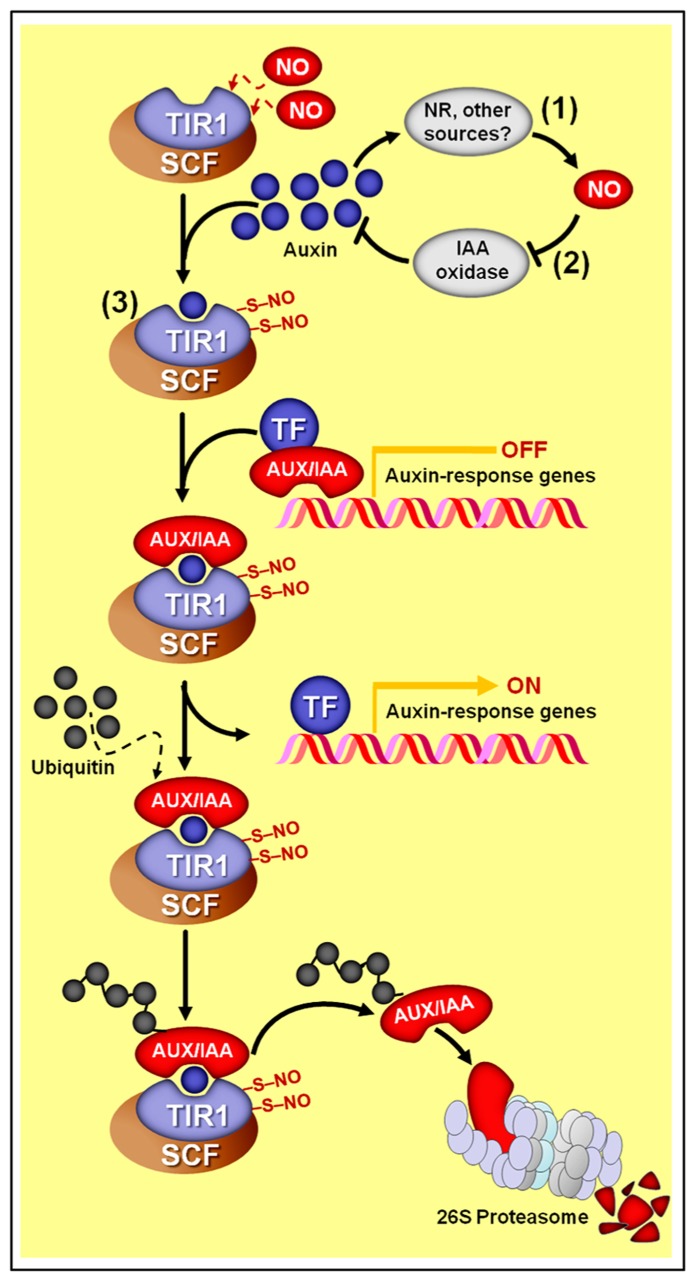
**Schematic representation of NO–auxin synergist interactions.** (1) Auxins stimulate NO production in several plant materials and experimental conditions. In most cases, nitrate reductase (NR) seems to be the main biosynthetic source of auxin-induced NO production. (2) In *M. truncatula* roots, NO promotes auxin accumulation by repressing its degradation via IAA-oxidase. (3) In *Arabidopsis*, NO might also positively impact auxin signaling since the auxin receptor TRANSPORT INHIBITOR RESPONSE 1 (TIR1) may undergo *S*-nitrosylation at cys-140 and cys-480, which promotes its interaction with AUXIN/INDOLE-3-ACETIC ACID (AUX/IAA) proteins. Subsequently, TIR1 marks AUX/IAA proteins to degradation through SCF-26S proteasome-mediated proteolysis, thereby de-repressing the transcription of auxin-regulated genes. Protein *S*-nitrosylation is represented by “–S–NO.”

Besides these impacts of auxin on NO production, recent studies have demonstrated that NO might also modulate auxin metabolism, transport, and signaling. For example, NO has been demonstrated to enhance root indole-3-acetic acid (IAA) levels in cadmium-treated *Medicago truncatula* seedlings by reducing its degradation via IAA oxidase activity (**Figure 4**), thereby positively impacting auxin equilibrium and ameliorating cadmium toxicity (Xu et al., 2010). In addition, pharmacological treatments and NO-overproducing mutants indicated that, at high concentrations, NO inhibits acropetal auxin transport in *Arabidopsis* roots by reducing the abundance of the auxin efflux protein PIN-FORMED 1 (PIN1) via a proteasome-independent post-transcriptional mechanism (Fernández-Marcos et al., 2011). This NO-dependent decrease in PIN1 protein levels and consequent disturbance in root auxin transport resulted in severe reductions in root meristem size and activity in primary roots due to a reduction in cell division and a promotion in cell differentiation, compromising the root apical meristem maintenance and primary root growth (Fernández-Marcos et al., 2011).

Finally, a direct influence of NO on auxin perception and signal transduction has also been suggested based on the recent demonstration that the auxin receptor protein TIR1 (TRANSPORT INHIBITOR RESPONSE 1) undergoes *S*-nitrosylation at two particular cysteine residues (cys-140 and cys-480) (Terrile et al., 2012). This *S*-nitrosylation of TIR1 seems to promote its interaction with AUXIN/INDOLE-3-ACETIC ACID (AUX/IAA) proteins, which are transcriptional repressors of genes associated with auxin responses (**Figure 4**). Being part of an E3 ubiquitin ligase complex, TIR1 marks AUX/IAA proteins to proteasome degradation, de-repressing the expression of auxin-dependent genes. Therefore, as a result, the increased TIR1–AUX/IAA interaction caused by TIR1 *S*-nitrosylation may facilitate AUX/IAA degradation via proteasome and subsequently promote auxin-dependent gene expression (Terrile et al., 2012). A possible impact of *S*-nitrosylation on the capacity of TIR1 to bind auxin could also be a possible outcome of this NO-dependent PTM, but further investigations are still required on this subject.

Furthermore, evidence indicates that nsHbs might also influence and modify the auxin signaling and action site by modulating the endogenous NO levels. Hunt et al. (2002), for example, detected a drastic modification in auxin-regulated root morphology and development in transgenic lines of *Arabidopsis* overexpressing class 1 nsHb, which could be interpreted as the result of changes in the content and/or bioactivity of NO in these plants.

### NO AND CYTOKININS INTERACTIONS

During the last few years, accumulating evidence has indicated complex and multilevel interactions between NO and cytokinins. Both synergistic and antagonistic interactions between NO and cytokinins have been described depending on the physiological response, plant species and experimental approach. Evidence implying a possible participation of NO in cytokinin signal transduction was first obtained during the accumulation of the red pigment betalaine in *Amaranthus caudatus* seedlings, which was shown to positively respond not only to cytokinins but also to NO gas or donors (Scherer and Holk, 2000). Since then, a number of studies have reported rapid and dose-dependent increases in NO production triggered by μM concentrations of cytokinins in both plant cell cultures (Tun et al., 2001; Carimi et al., 2005) and intact seedlings (Tun et al., 2008; Shen et al., 2012). In *Arabidopsis* seedlings, for instance, zeatin triggered increases in NO production within 3 min via a biosynthetic mechanism sensitive to arginine analogs and apparently independent of NR activity (Tun et al., 2008). However, other evidence revealed unchanged or even lower NO levels after cytokinin treatments or in mutant or transgenic plants with increased cytokinin production (Xiao-Ping and Xi-Gui, 2006; Romanov et al., 2008; Liu et al., 2013). Moreover, no obvious influence of exogenous application or depletion of NO has been observed on some early signaling events leading to the induction of primary cytokinin responses, such as the activation of cytokinin-responsive *Arabidopsis response regulator* (*ARR*)*5* promoter in seedlings (Romanov et al., 2008).

Examples of synergistic interaction between cytokinins and NO include the control of leaf senescence (Mishina et al., 2007), programmed cell death (PCD; Carimi et al., 2005), photosynthesis adaptability to drought stress (Shao et al., 2010), cell division, and differentiation (Shen et al., 2012), among others. Studies of the integrated influence of NO and cytokinins on plant senescence program have demonstrated that natural, dark- or dehydration-induced leaf senescence can be minimized by exogenous NO application (Cheng et al., 2002; Mishina et al., 2007). In addition, mutant or transgenic plants exhibiting decreased NO levels usually display precocious senescence in detached leaves and intact plants (Guo and Crawford, 2005; Mishina et al., 2007), which can sometimes be alleviated by exogenous cytokinin supplementation (Mishina et al., 2007). Although still limited in terms of current commercial application, this antisenescence trait of NO and cytokinins has been proven to extend post-harvest life of agronomically relevant fruits and vegetables (Leshem and Wills, 1998; Leshem et al., 1998; Leshem et al., 2001).

Further indicating a protective and antisenescence role of NO and cytokinins, Shao et al. (2010) reported increased NO levels during the cytokinin-induced photosynthetic adaptability to drought stress and described a good correlation between NO production and NR activity during this adaptive plant response to water limitation. In contrast, however, NOS-like-dependent increases in NO generation have been suggested to act as an intermediate during the acceleration of cell apoptosis induced by high cytokinin dosages since cell death was alleviated when cytokinins were supplied along with NOS inhibitors or NO scavengers to *Arabidopsis* cell cultures (Carimi et al., 2005).

The interaction between cytokinins and NO during the regulation of plant cell division has also been recently studied in more detail. Among other evidence, NO deficiency caused either by loss of the gene *NOA1* or due to NO scavenger treatments was demonstrated to result in severe inhibition of cytokinin-induced transcriptional activation of the cell cycle gene *CYCD3;1* (*CYCLIN-D3;1*) and the subsequent callus initiation from somatic plant tissues, implying that NO may act downstream of cytokinins in the control of plant cell mitotic cycles (Shen et al., 2012). In this study, roots of *Atnoa1* mutant were described as severely impaired in cytokinin-induced NO production and less sensitive to cytokinins than wild-type (WT) ones (Shen et al., 2012).

Contrary to the above described synergistic relationships between NO and cytokinins, literature data have also suggested an opposite interaction between these signaling molecules in some plant responses (Xiao-Ping and Xi-Gui, 2006). Studies conducted on epidermal strips of *Vicia faba* indicated that exogenous cytokinins efficiently reduced NO generation in guard cells exposed to the NO donor sodium nitroprusside (SNP) as well as promoted stomata reopening under dark condition due to the abolishment of the dark-induced increases in endogenous NO, which was interpreted as evidence of a potential scavenging action of cytokinins on the NO produced under these situations (Xiao-Ping and Xi-Gui, 2006).

**FIGURE 5:**
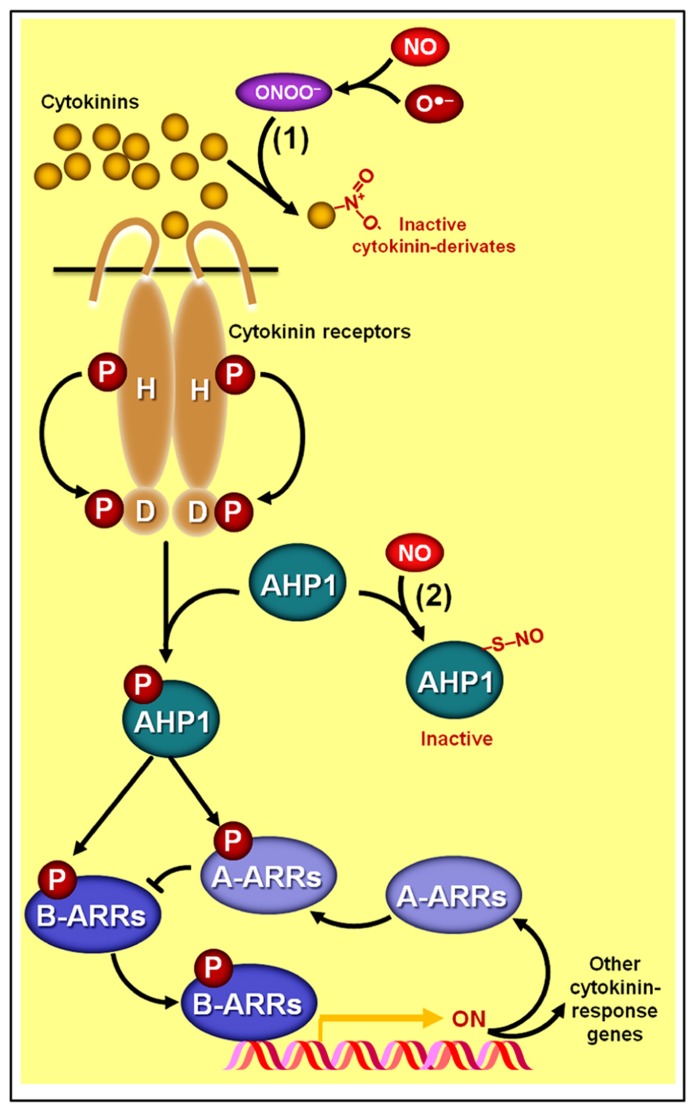
**Schematic representation of NO–cytokinin antagonistic interactions.** (1) Certain cytokinin species such as zeatin may chemically react with peroxynitrite (ONOO^-^), producing derivates with virtually no biological activity. (2) NO might also negatively impact cytokinin signaling since the protein HISTIDINE PHOSPHOTRANSFER PROTEIN 1 (AHP1), a key element in the phosphorelay mechanism involved in cytokinin transduction in *Arabidopsis*, may undergo *S*-nitrosylation at cys-115, rendering this protein incapable of transferring phosphoryl groups from the cytokinin receptors to the ARABIDOPSIS RESPONSE REGULATORs (ARRs). Protein *S*-nitrosylation and phosphorylation are represented by “–S–NO” and “P,” respectively.

Consistent with these results, Wilhelmová et al. (2006) also observed a negative correlation between endogenous cytokinin and NO levels in transgenic tobacco plants with either increased or decreased cytokinin levels. More recently, Liu et al. (2013) reported that cytokinins might intimately participate in NO catabolism since some cytokinin species, such as zeatin, can chemically react with peroxynitrite, leading to the production of cytokinin derivates with virtually no biological activity (**Figure 5**). Moreover, these authors verified that exogenous zeatin alleviates the severity of the phenotypes attributed to excessive NO levels in the *Arabidopsis* NO-overproducer *nox1* (*nitric oxide overexpression 1*) mutant, and this same ameliorative effect was observed when *nox1* plants were crossed with a cytokinin-overproducing mutant (Liu et al., 2013). Based on these biological and chemical data, Liu et al. (2013) postulated that these two signaling molecules (NO and cytokinins) might interact by modulating each other’s homeostatic levels and bioactivity (**Figure 5**). Such peculiar mechanism of interaction between cytokinins and NO, in which one of the substances directly interferes with the levels of another simply by a chemical combination of two molecules (**Figure 2C**), is quite different from the interaction at biosynthetic or signaling levels usually observed for other NO–phytohormone crosstalks (**Figures 2A, B**) and certainly deserves further attention.

Additionally, strong evidence indicating a direct impact of NO on the cytokinin signaling pathway has recently been uncovered (Feng et al., 2013). Besides corroborating previous observations that *Arabidopsis* mutant lines with excessive NO levels display more limited responsiveness to cytokinins, Feng et al. (2013) revealed that the phosphorelay mechanism central to the signaling transduction of this hormonal class can be severely impaired by the *S*-nitrosylation of a particular cysteine residue (cys 115) of the HISTIDINE PHOSPHOTRANSFER PROTEIN 1 (AHP1), hindering the transfer of phosphoryl groups from cytokinin receptors to AHP1 and subsequently to response regulators (ARRs; **Figure 5**). Confirming the importance of this NO-dependent post-translational protein modification for the cytokinin signal transduction, these authors have demonstrated that non-nitrosylatable mutation of AHP1 consistently relieved the inhibitory effect of NO on cytokinin responses whereas a nitrosomimetic mutation of this protein severely compromised cytokinin responses (Feng et al., 2013).

An additional, less direct way through which cytokinins might modulate NO levels in plant systems seems to rely on the regulatory effect of these hormones on the expression of nsHbs (Hunt et al., 2001; Ross et al., 2004; Bustos-Sanmamed et al., 2011). Cytokinin-triggered changes in the expression of certain nsHbs have been described for several plant models (Ross et al., 2004; Bustos-Sanmamed et al., 2011). Moreover, transgenic and mutant plants with altered levels of particular nsHb classes have frequently displayed alterations in plant responses typically controlled by cytokinins (Hunt et al., 2001; Wang et al., 2011). For instance, marked changes in shoot organogenesis and altered expression of genes associated with cytokinin perception and signaling have been observed in *Arabidopsis* lines silencing or overexpressing class 1 or class 2 nsHbs (Wang et al., 2011). In the transgenic lines overexpressing nsHbs, cytokinin feedback repressors (Type-A ARRs) were repressed, whereas cytokinin activators (Type-B ARRs) and receptors were stimulated (**Figure 3**), culminating in a higher sensitivity of the tissues to the cytokinin-induced shoot organogenesis (Wang et al., 2011). Unfortunately, NO content was not measured in these transgenic lines; therefore, a direct correlation between the higher responsiveness to cytokinins observed in nsHb overexpressing lines and their possibly lower NO levels could not be established.

### NO AND ABSCISIC ACID INTERACTIONS

Both important “stress-related” molecules, NO and ABA intensively crosstalk during certain signaling cascades triggered by environmental challenges, such as water limitation and UV-B radiation, which ultimately leads to the induction of plant adaptive responses, such as stomatal closure and antioxidant defenses (Neill et al., 2008a; Tossi et al., 2009; Hancock et al., 2011). During the induction of these plant stress responses, NO mainly acts as a downstream element in the ABA signaling pathway since the impairment in NO production or its removal from tissues usually decreases or even eliminates ABA responses while the inhibition of ABA production typically does not affect the induction of these responses by exogenous NO application. On the other hand, during the regulation of certain developmental events not directly linked to plant stress responses, such as seed dormancy breaking, NO seems to counteract ABA effects (Bethke et al., 2006; Lozano-Juste and Leon, 2010a, b), suggesting a certain level of specificity in the NO–ABA interaction mechanisms, which may depend on the physiological events under analysis (e.g., stomatal closure *versus* seed dormancy release) or even the type of plant cell, tissue, or organ considered (e.g., guard cell *versus *seed tissues).

**FIGURE 6:**
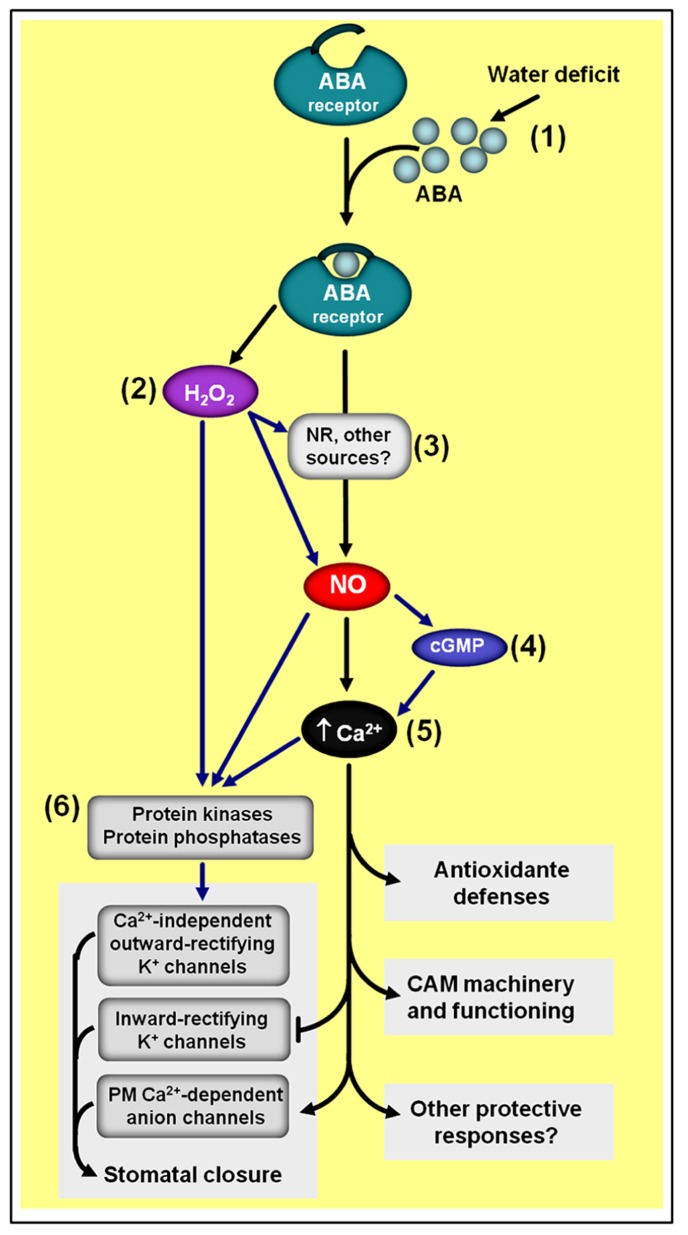
**Simplified schematic representation of NO–ABA interactions during defense responses to water shortage.** (1) Water deficiency usually increases endogenous ABA levels. (2) ABA-induced NO generation depends on hydrogen peroxide (H_2_O_2_) synthesis. (3) NR seems to be one of the main sources of ABA-induced NO production. (4) NO-triggered changes in cytosolic calcium (Ca^2+^) seem to involve cyclic guanosine monophosphate (cGMP). (5) The calcium/calmodulin system is a key downstream element of NO/ABA signaling. (6) Protein kinases and phosphatases are typical targets of H_2_O_2_, NO, and Ca^2^^+^/calmodulin during ABA-induced responses. Black arrows indicate signaling steps shared by all three drought responses considered in the scheme (i.e., stomatal closure, antioxidant defenses, and Crassulacean acid metabolism induction). Blue arrows indicate some steps currently described only for the regulation of stomatal closure and/or antioxidant defenses. ABA- and NO-independent signaling pathways are not represented in this****schematic representation.

In some cases, such as during the induction of stomatal closure (Neill et al., 2002; Desikan et al., 2004; Bright et al., 2006) and during the up-regulation of the gene transcription and activities of antioxidant enzymes (Zhang et al., 2007; Lu et al., 2009; Zhang et al., 2009), ABA-induced NO generation seems to depend on H_2_O_2_ synthesis, suggesting this ROS as a mediator in NO-dependent ABA responses (**Figure 6**). In addition, the calcium/calmodulin system and MAPKs have also being identified as downstream elements of NO signaling during the regulation of plant antioxidant defenses induced either by ABA or H_2_O_2_ (Zhang et al., 2007; Sang et al., 2008). Moreover, cGMP has also been demonstrated to participate in NO-dependent ABA signaling, apparently acting downstream of NO and upstream of cytosolic Ca^2^^+^ (**Figure 6**; Dubovskaya et al., 2011). Similarly, type 2C protein phosphatases (PP2Cs), which acts as negative regulators of ABA signaling, have also been suggested to play a role as putative crosstalk elements between ABA receptors and NO-mediated ABA signal transduction, possibly acting downstream of NO in the complex networks controlling ABA-triggered stomatal closure (Desikan et al., 2002).

Since the discovery that NO scavengers could reduce ABA-induced stomata closure in turgid leaves of different plant species (Garcia-Mata and Lamattina, 2002; Neill et al., 2002), intensive research has been dedicated to characterize the mechanisms underlying the interplay between these two molecules in guard cell signaling networks (reviewed in Neill et al., 2008a; Hancock et al., 2011; Simontacchi et al., 2013), leading to the identification of several NO targets during the ABA-induced guard cell responses. Among these targets, plasma membrane calcium-dependent anion channels and inward-rectifying K^+^ channels have been demonstrated to be activated and deactivated, respectively, by NO as a consequence of increases in guard cell cytoplasmatic Ca^2^^+^ levels (**Figure 6**) due to NO-triggered release of this anion from intercellular stores (Garcia-Mata et al., 2003).

Evidence for the involvement of protein phosphorylation upstream of intracellular calcium release has also been obtained, implicating protein kinases as additional targets of NO action within ABA-regulated guard cell signaling (Sokolovski et al., 2005). Moreover, NO has also been reported to directly modulate calcium-independent outward-rectifying K^+^ channels possibly by post-translationally modifying these channels or closely associated regulatory proteins (Sokolovski and Blatt, 2004). As a final consequence, this NO-dependent modulation of both Ca^2^^+^-dependent and Ca^2^^+^-independent ion channels at the plasma membrane of guard cells facilitates osmotic solute loss, thereby reducing guard cell turgor and promoting stomatal closure.

It is worth mentioning that NO has been suggested to play a role as a second messenger shared by multiple hormonal signaling cascades involved in the intricate guard cell network responsible for coordinating stomatal movement in higher plants, mediating not only the ABA signal but also ethylene (Liu et al., 2010), salicylic acid (SA; Hao et al., 2010), methyl jasmonate (Saito et al., 2009), auxin, and cytokinins (Xiao-Ping and Xi-Gui, 2006). Curiously, though, NO apparently is not an absolute requirement during the ABA signaling cascades leading to stomatal closure (Ribeiro et al., 2009) or the inhibition of light-induced stomatal opening (Yan et al., 2007; Yang et al., 2008); therefore, the existence of both NO-dependent and NO-independent pathways in ABA-induced guard cell responses is currently being suggested. Of course, more studies are clearly needed to better characterize a possible integrative, but apparently non-essential, role of NO during the regulation of stomatal movements by distinct environmental and hormonal stimuli.

At least in bromeliads, NO and ABA also seem to intensively interact to control Crassulacean acid metabolism (CAM) expression (Freschi et al., 2010; Mioto and Mercier, 2013), which, in turn, facilitates the survival of these plants under water- and nutrient-limited environments. As during the regulation of stomatal movements, NO apparently acts downstream of ABA and upstream of cytosolic calcium in the ABA-dependent signaling cascade leading to the up-regulation of the CAM machinery (**Figure 6**), and does not participate in the ABA-independent pathway also responsible for the regulation of this plant stress response (Freschi et al., 2010). The regulation of CAM expression in bromeliads as well as the control of stomata movements in *Arabidopsis* seem to have NR activity as the main source of the ABA-induced NO production (Desikan et al., 2002; Freschi et al., 2010).

While a number of pharmacological and genetic studies have reported higher endogenous NO levels following increases in plant tissue ABA concentration (i.e., NO action downstream of ABA; Zhang et al., 2009), NO-triggered changes in ABA biosynthesis and catabolism (i.e., NO action upstream of ABA) have rarely been described. In one of the few examples, Liu et al. (2009) reported that during the seed dormancy breaking in *Arabidopsis*, a rapid accumulation of NO in the endosperm layer preceded a decrease in ABA concentration, which was associated with a pronounced rise in the transcript and protein levels of the ABA 8′-hydroxylase CYP707A2, a key enzyme in ABA catabolism. Moreover, exogenous NO and the NO scavenger carboxy-PTIO (cPTIO), respectively, induced and impaired *CYP707A2 *transcript accumulation during the imbibition period (Liu et al., 2009), further suggesting that the promotive effect of NO on seed dormancy break might indeed be associated with a stimulation of ABA catabolism.

In addition to modulating ABA catabolism, NO has also been described to affect the sensitivity of plant cells to ABA (Bethke et al., 2006; Lozano-Juste and Leon, 2010a, b). Bethke et al. (2006) reported that the NO donor SNP enhanced germination of dormant *Arabidopsis* seeds by decreasing the seed sensitivity to exogenous ABA. More recently, genetic evidence supporting this inhibitory effect of NO on ABA sensitivity was obtained by Lozano-Juste and Leon (2010a), b, who observed that the depletion of endogenous NO levels resulting from the generation of the *nia1,2noa1-2* *Arabidopsis* triple mutant clearly led to ABA hypersensitivity. Among other features, this triple mutant displayed enhanced seed dormancy, decreased seed germination, and reduced seedling establishment in the presence of exogenous ABA, reinforcing the hypothesis that NO production during seed germination and initial seedling development counteracts the ABA inhibitory effects on these events. Interestingly, this ABA hypersensitivity continued through the post-germinative vegetative development of this triple mutant, as evidenced by the presence of increased expression of ABA-responsive genes, extreme drought resistance phenotype as well as higher responsiveness to ABA during stomatal closure (Lozano-Juste and Leon, 2010a, b). Curiously, dehydration- and ABA-dependent stomatal closure normally occurred in the presence of undetectable NO production in guard cells, corroborating the existence of a NO-independent pathway in this guard cell response (Ribeiro et al., 2009). Whether NO exerts its effects directly on ABA receptors or on some downstream element of ABA signaling cascade is obviously an important question that remains to be answered.

### NO AND GIBBERELLINS INTERACTIONS

Nitric oxide has also been reported to influence several plant developmental events in which GAs play crucial roles, such as seed germination, hypocotyl elongation, acquisition of photomorphogenic traits, primary root growth, reorientation, and growth of pollen tubes, among others (Beligni and Lamattina, 2000; Prado et al., 2008; Tonón et al., 2010; Leon and Lozano-Juste, 2011); however, thus far, the actual interaction between NO and GAs has been described for only a limited number of these physiological events. In fact, most of our current knowledge of the mechanisms underlying the interplay between GAs and NO is restricted to the regulation of seed germination (Beligni et al., 2002; Bethke et al., 2007) and the inhibition of hypocotyl elongation during seedling de-etiolation (Leon and Lozano-Juste, 2011). During the control of these responses, NO has been described to act upstream of GA (Bethke et al., 2007), regulating both GA biosynthesis and perception/transduction (Leon and Lozano-Juste, 2011).

**FIGURE 7:**
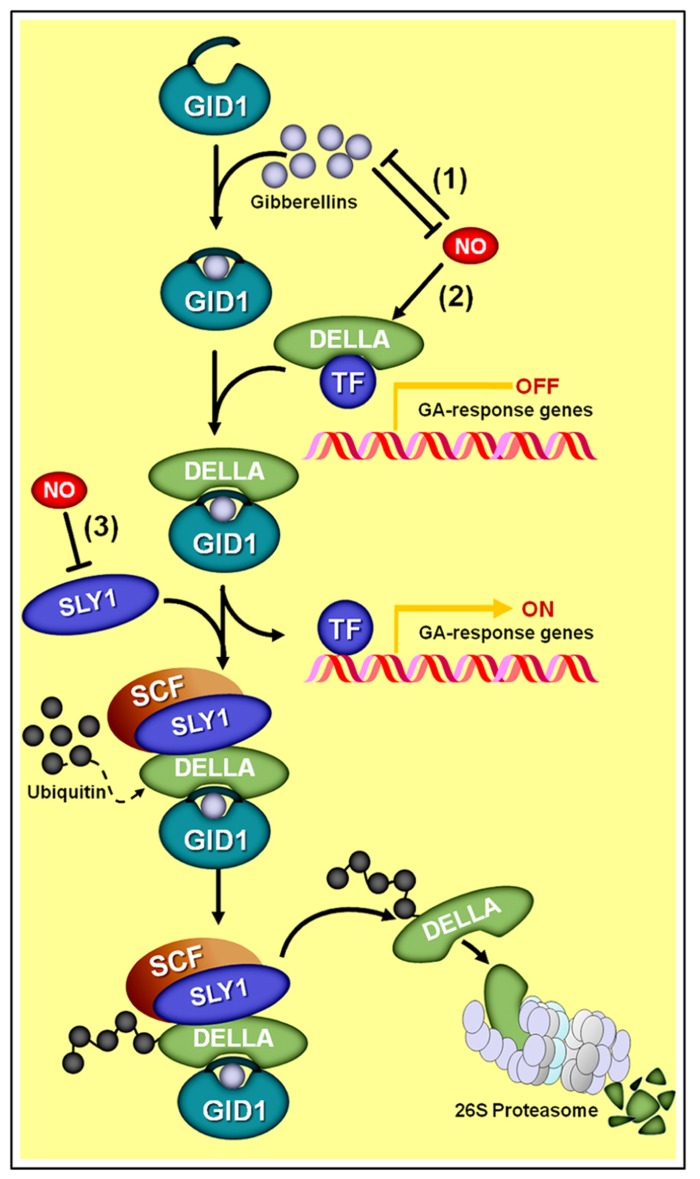
**Schematic representation of NO–gibberellin antagonistic interactions.** (1) A mutual antagonism controls the endogenous levels of NO and gibberellins in *Arabidopsis* seedlings. (2) Additionally, NO negatively influences GA signaling by promoting the accumulation of DELLA proteins, whose presence represses the transcription of GA-regulated genes. Since the degradation of DELLAs through SCF-26S proteasome-mediated proteolysis depends on the interaction of these proteins with the complex formed by active gibberellin molecules associated with the receptor GA INSENSITIVE DWARF1 (GID1) and the E3 ubiquitin ligase SLEEPY1 (SLY1), the NO-driven increase in DELLAs and reduction in SLY1 abundance (3) negatively impacts the transduction of the GA signal.

A certain level of antagonism between NO and GAs has been observed for most of the physiological processes in which both of these signaling compounds participate. A mounting body of evidence has indicated that DELLA proteins apparently represent a key crosstalk component between GA and NO signaling interactions (**Figure 7**; Leon and Lozano-Juste, 2011). DELLA proteins are a relatively small family of transcriptional regulators notably important for the integration of diverse hormonal signals, such as GAs, ethylene, jasmonate (JA), and ABA (Achard et al., 2003; Gao et al., 2011; Ross et al., 2011). During GA signaling transduction, for instance, the hormonal molecules interact with GA INSENSITIVE DWARF1 (GID1) receptors, which, in turn, binds a DELLA protein and subsequently directs the GA–GID1–DELLA complex to the E3 ubiquitin ligase SLEEPY1 (SLY1), thereby promoting DELLA degradation at the proteasome (**Figure 7**). Given that DELLAs mainly act by repressing the transcription of GA-regulated genes, the perception and transduction of the GA signal leads, as a final result, to a decrease in DELLA concentration into the cell and a consequent induction of GA-responsive genes.

Interestingly, recent studies have indicated that NO triggers the opposite effect on cellular DELLA concentration, promoting the accumulation of this protein and a consequent negative impact on GA signal transduction (**Figure 7**). Essentially, this NO-driven DELLA accumulation can be interpreted as a reduction in tissue sensitivity to GA since a larger number of GA–GID1–DELLA complexes will need to be formed in order to mark an adequate quantity of DELLA proteins for proteasome degradation, thereby leading to a satisfactory level of transcriptional de-repression of GA-regulated genes. This differential effect of NO and GAs on DELLA regulation might account, at least in part, for the antagonism observed between these two signaling compounds during the regulation of physiological processes, such as hypocotyl elongation (Leon and Lozano-Juste, 2011) and primary root growth (Fernández-Marcos et al., 2012) in *Arabidopsis*.

In addition, studies performed on *nia1,2noa1-2* seedlings revealed that this NO-deficient mutant presents defective DELLA accumulation associated with an up-regulation of the E3 ubiquitin ligase SLY1 (**Figure 7**), resulting in increased GA sensitivity and deficient de-etiolation under red light (Leon and Lozano-Juste, 2011). Further emphasizing the potential role for DELLAs in the GA–NO antagonistic interactions, exogenous NO was also demonstrated to induce the accumulation of GA-regulated DELLA proteins (Leon and Lozano-Juste, 2011), very likely by negatively regulating the GID1–SLY1 system of DELLA tagging for degradation (**Figure 7**). However, as pointed out by Leon and Lozano-Juste (2011), the regulation of DELLA turnover and activity may represent the main but not the only target for NO action in regulating plant growth and other GA-mediated developmental responses since DELLA-independent mechanisms might also be implicated.

Besides the negative action of NO on GA signaling network, a mutual antagonism controlling the endogenous levels of these two signaling molecules has also recently been proposed (**Figure 7**) (Leon and Lozano-Juste, 2011). Supporting this suggestion, etiolated seedlings of the GA-deficient *Arabidopsis* mutant *ga1-3* have been shown to exhibit NO levels significantly higher than those observed in the WT genotype. Moreover, both *ga1-3* mutant and WT seedlings showed reduced NO levels after GA_3_ supplementation, thereby suggesting that GAs negatively modulates NO production (Leon and Lozano-Juste, 2011). On the other hand, WT *Arabidopsis* seedlings treated with SNP presented a significant reduction in endogenous GA levels (Leon and Lozano-Juste, 2011). Based on a detailed analysis of the expression of *Arabidopsis* genes involved in GA biosynthesis* (GA20oxidase* and *GA3oxidase*) and catabolism (*GA2oxidase)*, *GA20ox3* was identified as the only gene significantly up-regulated in the NO-deficient *nia1,2noa1-2 *mutant and down-regulated in NO-treated WT seedlings (Leon and Lozano-Juste, 2011).

Under certain circumstances, however, NO seems to play a stimulatory rather than inhibitory role in the GA biosynthetic machinery (Bethke et al., 2007). Exemplifying such a synergist relationship, Bethke et al. (2007) reported that NO generation was required for the transcription of two* GA3oxidase* genes (*GA3ox1* and *GA3ox2*) during the *Arabidopsis* seed dormancy breaking. Another indication of the positive interaction between GA and NO has recently been reported in wheat roots, for which the SNP-induced apical growth was associated with increased GA_3_ levels (He et al., 2012).

Apart from the above-mentioned evidence of NO acting upstream of GA, a certain level of uncertainty remains as to whether NO and GA actually share a common signaling route or just act through parallel, independent cascades during the regulation of some plant responses. During seed dormancy breaking, for instance, although there is virtually no doubt that both of these signal molecules promote germination in a number of species (Giba et al., 1998; Beligni and Lamattina, 2000; Kopyra and Gwozdz, 2003), whether and how NO and GA interact during this process still needs further characterization.

In fact, whereas a mounting body of evidence indicates that NO selectively interferes in some specific GA-induced events associated with the seed germination process, such as the longevity of cereal aleurone cells (Beligni et al., 2002), transcription of Myb transcription factor (GAMYB), and amylase synthesis (Wu et al., 2013), for some other responses associated with the germination process, no indications of additive or antagonistic responses have been found when both GA and NO were exogenously applied (Zhang et al., 2005). In addition, a rapid burst in NO production has been detected during early seed germination (Simontacchi et al., 2004), which has been speculated to be temporally dissociated from the action of GAs at later stages of seed germination (Zhang et al., 2005).

Regardless of whether or not NO and GA share a common signaling cascade during seed dormancy breaking, the stimulation of seed germination by either of these substances can be blocked by sufficiently high concentrations of ABA (Bethke et al., 2004, 2006; Sarath et al., 2006; Dong et al., 2012). Considering that NO may stimulate germination not only by breaking seed dormancy but also by alleviating the influence of environmental factors inhibitory to the germination process (Bethke et al., 2007), a NO-hormonal network much more complex than the interaction between NO, GA, and ABA might possibly be involved in the regulation of this critically important step in the plant life cycle.

### NO AND ETHYLENE INTERACTIONS

A significant number of the currently available reports on the interaction between NO and ethylene suggest an antagonistic relationship between these two gaseous molecules (Leshem et al., 1998; Lamattina et al., 2003; Manjunatha et al., 2010). The first and presently most explored plant phenomenon in which NO was demonstrated to counteract ethylene production and action is the control of fruit ripening and the regulation of leaf and flower senescence (Leshem et al., 1998; Manjunatha et al., 2010). For these responses, ethylene has long been identified as a key promotive signal, and a large number of reports indicate that the production and perception mechanisms of this plant hormone are under strict regulation, depending not only on the plant developmental program but also on a number of environmental factors (Grbić and Bleecker, 1995; Fischer, 2012). Additional studies revealed that exogenous application of NO, either by direct fumigation or by means of NO-releasing chemicals, delays senescence of both vegetative and reproductive organs by negatively regulating a number of elements involved in ethylene production (Leshem and Haramaty, 1996; Leshem et al., 1998; Wills et al., 2000; Zhu et al., 2006; Liu et al., 2007; Manjunatha et al., 2010, 2012). Corroborating this pharmacological evidence, measurements of ethylene and NO emission during either fruit ripening (Leshem et al., 1998; Leshem and Pinchasov, 2000) or plant senescence (Magalh a, 2000; Corpas et al., 2004) revealed an opposite trend between these gases, in which ethylene production increases, whereas NO levels decrease during the induction and establishment of these processes.

**FIGURE 8:**
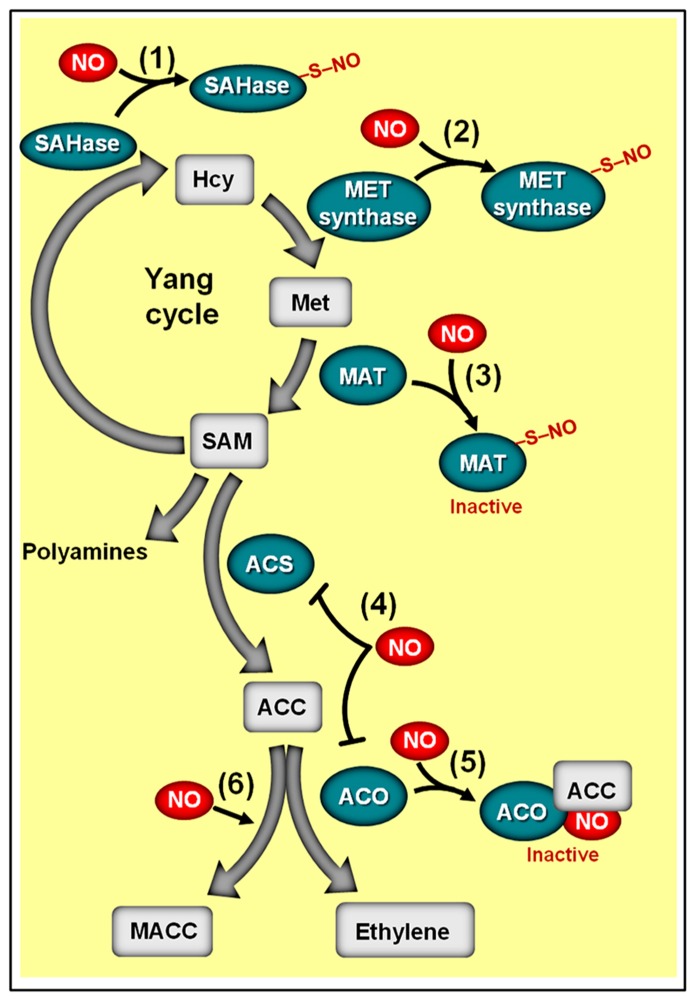
**Schematic representation of NO-ethylene antagonistic interactions.** The methylmethionine cycle enzymes adenosyl homocysteinase (SAHase) (1) and methionine synthase (MET synthase) (2), whose activities are responsible for the production of homocysteinase (Hcy) and methionine (Met), respectively, may undergo *S*-nitrosylation. (3) Additionally, the activity of the *Arabidopsis* methionine adenosyltransferase 1 (MAT1) can be suppressed by *S*-nitrosylation, thereby repressing the conversion of methionine (Met) to *S*-adenosyl methionine (SAM). (4) In ripening climacteric fruits, NO has been shown to inhibit the transcript levels of 1-aminocyclopropane 1-carboxylic acid (ACC) synthase (ACS) and/or ACC oxidase (ACO). (5) NO can also inhibit ACO activity by directly binding this enzyme, resulting in the ACO–NO binary complex, which subsequently originates a ternary stable complex ACO–NO–ACC. (6) NO-driven accumulation of non-volatile ACC metabolite 1-malonyl aminocyclopropane-1-carboxylic acid (MACC) has also been reported. Biosynthetic enzymes are represented with green ovals and metabolic substrates and products with gray rectangles. Protein *S*-nitrosylation is represented by “–S–NO.” Note that the impact of *S*-nitrosylation on the activities of SAHase, MET synthase, and ACS remains to be determined.

Recent studies have revealed that the inhibition of fruit ethylene production by NO may be attributed to a reduction in the transcript level and/or activity of key ethylene biosynthetic enzymes (Manjunatha et al., 2010). In vegetative and reproductive plant tissues, ethylene production depends on the conversion of the *S*-adenosyl methionine (SAM), derived from “Yang cycle,” into the immediate ethylene precursor 1-aminocyclopropane 1-carboxylic acid (ACC) through ACC synthase (ACS) activity (**Figure 8**). The ACC formed may be subsequently converted to ethylene due to the activity of a second enzyme, the ACC oxidase (ACO; Yang and Hoffman, 1984). Since the abundance of ACC, ACS, and ACO in plant tissues represents a critical aspect for determining ethylene production rates (Barry et al., 1996; Barry et al., 2000), an inhibitory effect of NO on any of these elements can be expected to be an efficient mechanism for down-regulating ethylene synthesis.

As revealed by a series of studies on climacteric fruits, exogenous NO indeed has the capacity to modulate both the transcription and the activity of both ACS and ACO (**Figure 8**), consequently impacting not only the levels of ethylene production but also the accumulation of ACC (Manjunatha et al., 2010). In tomato fruits, for instance, although the expression of all ACS homologs remained virtually unchanged following NO fumigation, the transcript abundance of ACO genes, such as *LeACO1*, *LeACOH2*, and *LeACO4*, and the levels of ethylene emission were reduced and/or delayed when NO was applied before the start of the ripening process (Eum et al., 2009). In banana fruits, on the other hand, NO negatively impacted the expression of both ACS and ACO homologs, leading to a reduction in ACO activity and ethylene emission as well as an accumulation of ACC (Cheng et al., 2009).

Apart from controlling the transcript levels of ACS and ACO, NO may also regulate ACS activity via *S*-nitrosylation (Abat and Deswal, 2009) and influence ACO activity by a mechanism involving the direct binding of NO to the enzyme, resulting in the ACO–NO binary complex, which is then chelated by ACC to produce the ternary stable complex ACO–NO–ACC (**Figure 8**) (Tierney et al., 2005; Zhu et al., 2006; Manjunatha et al., 2010). Currently, the impacts of *S*-nitrosylation on ACS activity remain uncharacterized, and the occurrence of the ACO–NO–ACC ternary complex is exclusively described during *in vitro* studies conducted on recombinant ACO (Tierney et al., 2005); therefore, the actual *in vivo* implications of such regulatory mechanisms still need further elucidation. Nevertheless, the hypothetical formation of an ACO–NO–ACC complex has already been inferred as possibly responsible for the reduction of ACO activity in climacteric peach (*Prunus persica*) fruits subjected to NO fumigation, which resulted in a concomitant decrease in ethylene emission and accumulation of ACC (Zhu et al., 2006). In this specific case, the NO-induced reduction of ACO activity was accompanied by an increment in the accumulation of the non-volatile ACC metabolite 1-malonyl aminocyclopropane-1-carboxylic acid (MACC; **Figure 8**), which was interpreted as a secondary effect of NO during the ripening of these fruits (Zhu et al., 2006).

Besides stimulating the irreversible conversion of ACC into MACC, NO may also negatively impact the turnover of SAM, which is the main precursor molecule for ACC synthesis. Supporting this assumption, proteomic analysis of *Arabidopsis* plants revealed that the methylmethionine cycle enzymes adenosyl homocysteinase (SAHase), methionine synthase (MET synthase) and methionine adenosyltransferase (MAT, also known as SAM synthase), whose activities are responsible for the production of homocysteinase (HCY), methionine (Met), and SAM, respectively, may undergo *S*-nitrosylation (**Figure 8**). In addition, similar analyses conducted on GSNO-treated protein extracts of *Kalanchoe pinnata* (Abat et al., 2008) and *Brassica juncea* (Abat and Deswal, 2009) also identified cobalamin-independent MET synthases as a common target of *S*-nitrosylation. Whereas the influence of *S*-nitrosylation on the activities of SAHase and MET synthase has yet to be determined, a detailed study conducted by Lindermayr et al. (2006) revealed that the activity of MAT1, one of the three *Arabidopsis* MAT isoforms, is indeed suppressed via *S*-nitrosylation at cys-114, having as a logical consequence the depletion of the SAM pool and a reduction in ethylene production. Curiously, the study conducted by Lindermayr et al. (2006) was the first detailed characterization of *S*-nitrosylation in plant systems, opening up a new window of opportunities for accessing the actual relevance of this NO-dependent post-translational regulatory mechanism in plant signaling.

In contrast to the above-mentioned evidence of an antagonistic relationship between NO and ethylene during the maturation, senescence, and abscission of plant organs, a number of reports have also indicated that NO donors, such as SNP, might sometimes stimulate, rather than negate, ethylene production in certain plant materials, such as non-senescent leaf tissues of *Arabidopsis*, tobacco, and maize (Magalhães et al., 2000; Ederli et al., 2006; Wang et al., 2006; Mur et al., 2008; Ahlfors et al., 2009) and apple embryos (Gniazdowska et al., 2007). In tobacco leaves, for instance, SNP infiltration has been show to stimulate ACS expression (Ederli et al., 2006; Mur et al., 2008), whereas in *Arabidopsis* roots the application of GSNO positively impacted the transcript levels of not only ACS but also other key ethylene biosynthetic enzymes, such as SAM synthetases, ACOs, and 5-methylthioribose kinase (MTK; Garcia et al., 2011). Further emphasizing a stimulatory influence of NO on ethylene biosynthesis, ethylene production is usually elevated when the NO accumulation is promoted via suppression of nsHbs gene expression (Manac’h-Little et al., 2005; Hebelstrup et al., 2012). Similarly, the increased NO production observed in transgenic tobacco lines expressing mammalian NOS were accompanied by a higher expression of ACO and some other ethylene-related genes (Chun et al., 2012). Moreover, a concomitant increase in both ethylene and NO emission has been consistently observed both in tobacco leaves undergoing bacterially triggered hypersensitive response (Mur et al., 2012b) and in *Arabidopsis* and cucumber (*Cucumis sativus*) roots subjected to Fe deficiency (Garcia et al., 2011).

Besides these indications of a positive influence of NO on ethylene production, some data also seem to support a stimulatory role of ethylene on NO production under certain circumstances (Garcia et al., 2011). Earlier in the research of NO–ethylene interaction in plants, Leshem and Haramaty (1996) reported that exogenous ACC induced significant increases in both ethylene and NO emission in pea (*Pisum sativum*) leaves. More recently, Garcia et al. (2011) have also detected increased NO levels in the root subapical region of *Arabidopsis* and cucumber plants exposed to ACC. In addition, these authors reported that inhibitors of ethylene biosynthesis and action completely abolished the increases in NO levels in roots of plants subjected to Fe deficiency. In contrast, ethylene supplementation or depletion, respectively, repressed and promoted NO production during the abscission of mature olive fruits (Parra-Lobato and Gomez-Jimenez, 2011), which apparently indicates that under certain circumstances ethylene may negatively, rather than positively, impact the endogenous NO levels. In agreement with this, ethylene has sometimes been shown to induce class 1 nsHbs (Qu et al., 2006; Bustos-Sanmamed et al., 2011), which in turn may lead to reductions in tissue concentration of NO.

Surprisingly, the possible influence of NO on ethylene signal transduction elements has remained virtually unexplored, both during antagonistic (e.g., fruit ripening and leaf senescence) and synergistic (e.g., plant defense to biotic stresses and Fe deficiency) interactions between these signaling substances. Therefore, it is currently unknown whether NO might regulate the transcripts levels or activities of receptors, signal transduction proteins and/or transcription factors involved in ethylene signaling, which would very likely impact the sensitivity of the plant tissues to this plant hormone. In one of the few studies on this line, Niu and Guo (2012) demonstrated that the dark-induced early senescence phenotype of the *Arabidopsis* NO-deficient mutant *noa1 *was suppressed by mutation in ETHYLENE INSENSITIVE 2 (EIN2) and indicated that this protein might act downstream of NO signaling, possibly playing a key role as a crosstalk point between ethylene and NO signaling cascades.

### INTERACTIONS BETWEEN NO AND OTHER PLANT HORMONES

Besides interacting with the five “classical” phytohormone classes, NO has also been reported to crosstalk with other plant hormones, including JAs, SA, polyamines, and brassinosteroids. Some of these interactions, such as the interplay between NO, SA, and JA in plant defense responses, have been investigated in great detail, uncovering impressively complex NO–phytohormone interaction networks. A detailed discussion about these interactions is beyond the scope of the present work; instead, just some brief, general comments, and examples of these NO–phytohormone crosstalks will be provided below.

**FIGURE 9:**
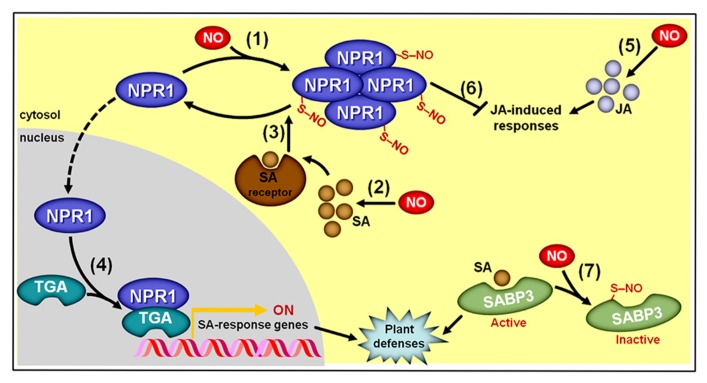
**Simplified schematic representation of NO, salicylic acid (SA), and jasmonic acid (JA) interactions during plant responses to biotic challenges.** (1) *S*-nitrosylation of NON-EXPRESSER OF PATHOGENESIS-RELATED GENE1 (NPR1) at cys156 promotes its oligomerization and permanence in the cytosol. (2) NO stimulates SA biosynthesis. (3) Oligomeric NPR1 is denitrosylated following its interaction with SA receptors, which promotes the formation of monomeric NPR1. (4) Monomeric NPR1 translocates to the nucleus, where this protein binds TGA-class transcription factors, which subsequently activate promoters of SA-responsive genes. (5) NO also stimulates JA biosynthesis. (6) Cytosolic, oligomeric NPR1 represses JA-triggered responses. (7) At late stages of bacterial infection, *S*-nitrosylation of SA-binding protein 3 (SABP3) at cys280 represses its SA binding capacity, thereby promoting a negative feedback loop during the defense signaling pathway. Protein *S*-nitrosylation is represented by “–S–NO.”

As recently reviewed by Yu et al. (2012) and Mur et al. (2013), during the induction of plant defense responses against biotic challenges, NO positively impacts the production of both SA and JA (Feechan et al., 2005; Chun et al., 2012; Mur et al., 2012b) and, at the same time, NO modulates SA signaling by controlling the oligomerization status of the translational activator NON-EXPRESSER OF PATHOGENESIS-RELATED GENE1 (NPR1) via *S*-nitrosylation at cys156 (Tada et al., 2008; **Figure 9**). *S*-nitrosylation of NPR1 facilitates its oligomerization (Tada et al., 2008) and permanence in the cytosol (Fu et al., 2012), where it may interact with SA receptors (NPR3/4). Following such interaction with NPR3/4, the *S*-nitrosylated cys156 of NPR1 is reduced (Tada et al., 2008), promoting NPR1 monomer formation and its consequent migration to the nucleus, where this protein may interact with several TGA-class transcription factors that subsequently activate promoters of SA-responsive genes (Mur et al., 2012b; **Figure 9**). In contrast, the presence of the *S*-nitrosylated, oligomeric form of NPR1 in the cytosol facilitates the repression of JA-triggered responses (Spoel et al., 2003). Consequently, this NO-dependent PTM of NPR1 seems to play a key integrative role during the hormonal signaling cascades leading to coordinated plant immunity responses (Yu et al., 2012; Mur et al., 2013). In parallel, *S*-nitrosylation of SA-BINDING PROTEIN 3 (SABP3) at cys280, which takes place during late stages of bacterial infection, represses its capacity to bind SA, and antagonizes the expression of plant immunity responses (Wang et al., 2009), thereby representing a negative feedback loop apparently essential for the correct regulation of SA-modulated plant defense against biotic challenges (**Figure 9**).

Accumulating evidence indicates that NO might also mediate both developmental and stress responses induced by polyamines (Wimalasekera et al., 2011). Briefly, very rapid NO production has been observed in plant tissues exposed to mM concentration of polyamines (Tun et al., 2006), which has sometimes been interpreted as an indication of a potential NO biosynthetic pathway involving the catabolism of these plant hormones (Wimalasekera et al., 2011). Given the absence of a lag phase between the application of polyamines and the rise in NO endogenous levels (Tun et al., 2006), it is currently assumed that these hormones might be directly converted to NO by the action of one or more enzymes, whose identities are yet to be determined (Wimalasekera et al., 2011). So far, it is only known that the polyamine-induced NO production can be quenched by mammalian NOS inhibitors and is not affected in *Arabidopsis* NR-deficient mutants (Tun et al., 2006; Wimalasekera et al., 2011). Whether polyamines act as substrates, cofactors, or signals for promoting NO synthesis also needs to be better determined; therefore, monitoring the formation of ^15^NO from isotopic-labeled polyamines in plant tissues or extracts seems an important experiment in future studies. A possible influence of NO on polyamine metabolism has been demonstrated in some studies (Fan et al., 2013) but not in others (Arasimowicz-Jelonek et al., 2009); consequently, this topic still deserves further investigation.

Considering that polyamines and ethylene share SAM as a common precursor, all the basic NO-dependent mechanisms controlling the SAM pools discussed earlier in this review (**Figure 8**) might also indirectly affect polyamine synthesis in plants. In addition, we also need to keep in mind that L-arginine is substrate for the production of polyamines, via arginase and arginine decarboxylase activities, as well as NO, via NOS-like activities; therefore, the availability of this particular amino acid might also influence NO/polyamine connections in plants and other organisms. In mammals, for instance, the occurrence of an arginine switch, in which NOS and arginase compete for arginine, seems to be supported by a great deal of experimental evidence (Satriano, 2004). In parallel, literature data in the animal field also indicates that polyamines such as spermidine and spermine influence NO production via NOS activity during diverse physiological responses (Guerra et al., 2006), which may represent an important source of information to guide current and future research on the NO and polyamine interactions in plants. The polyamine precursor agmatine, for example, has been demonstrated to act either as an alternative substrate or a competitive inhibitor of mammalian NOS, depending on the isoform or physiological process taken under consideration (Satriano, 2003; Raghavan and Dikshit, 2004), thereby indicating a possible role for this compound as an endogenous regulator of NO generation in mammals. In plants, the ameliorative effects of both polyamines and NO under stressful conditions (Arasimowicz-Jelonek et al., 2009; Wimalasekera et al., 2011; Gupta et al., 2013) might represent an important driving force to stimulate further studies on the interaction between these critically important signaling compounds.

Generation of information about whether and how NO and brassinosteroids interact has only recently begun (Zhang et al., 2011; Tossi et al., 2013). In one of these studies, Zhang et al. (2011) demonstrated that nM concentrations of brassinosteroids promoted rapid increases in the NO levels of leaf mesophyll cells, which together with some other evidence allowed the authors to place NO as a possible intermediate in the brassinosteroid-induced ABA biosynthesis in maize leaves. More recently, Tossi et al. (2013) reported that NR and NOS-like activities are probably involved in the brassinosteroid-induced NO production in *Arabidopsis* and that NO very likely mediates brassinosteroid-triggered modifications in plant root architecture.

## SOME CONCLUSIONS AND MANY UNANSWERED QUESTIONS

Despite the methodological difficulties and conceptual complexity intrinsically involved in the elucidation of the exact mechanisms responsible for interconnecting plant hormones and NO signaling during the coordination of plant metabolism and development, some cutting edge insights into the NO–phytohormone crosstalks have recently been achieved.

Many downstream and upstream components of the NO signaling cascades have been identified, and NO-dependent PTMs, notably *S*-nitrosylation, have emerged as critical mechanisms controlling key elements involved in plant hormone production and signaling. As highlighted in the course of this review, by chemically modifying these hormone-related proteins, NO may modify plant hormone metabolism and signaling at multiple, diversified levels. The identification and functional analysis of the protein targets of NO-dependent PTMs and whose action determines the delicate hormonal homeostasis in plants has been, and will probably continue to be, an approach of upmost relevance in NO–phytohormone studies.

Currently, the physiological relevance of NO-dependent chemical modifications of phytohormone-related proteins has been poorly investigated *in planta*; therefore, this remains a rich area for future investigation. Clarifying how these NO-triggered PTMs, particularly *S*-nitrosylation and tyrosine nitration, actually control protein activity, subcellular localization as well as protein–protein, protein–DNA, protein–cofactors, or even protein–hormone binding capacity will inexorably involve the use of a wide range of experimental strategies and methodological approaches, some of which are currently available (e.g., overexpression of modified proteins in mutant genetic backgrounds) and others yet to be developed. Since some proteins are targets of multiple NO-dependent PTMs, sometimes even involving different types of these chemical modifications (e.g., both *S*-nitrosylation and nitration; Lozano-Juste et al., 2011; Astier et al., 2012), it would be enlightening to determine the impacts of concomitant NO-triggered modifications on the same protein.

Moreover, characterizing how these target proteins are chemically modified by NO and NO-derivates at the right time and place seems to be another promising area of progress in NO–phytohormone interactions. Addressing this question inevitably implies dealing with several critical aspects of the NO physiology that still require further elucidation. Firstly, the basic mechanisms responsible for NO production, removal, and transport in plants continues to represent a critical impediment for advances in the clarification of how NO levels are temporally and spatially controlled by plant hormones and other stimuli. A fine-tuned equilibrium between NO production and removal (e.g., biosynthesis *versus* degradation, conjugation *versus* deconjugation) might possibly exist to determine both the localization and the concentration of NO and NO-derivates within the plant cells. Secondly, given the impressive diversity of target proteins, which are ubiquitously distributed within the plant cells, the existence of a certain subcellular compartmentation in NO production and action is an assumption that urgently needs to be investigated in greater detail. Moreover, a concentration-dependent action mode for NO has also been proposed (Mur et al., 2013), in which distinct responses may be triggered depending on the abundance of this free radical. Obviously, the development of more sensitive and specific means to determine the subcellular localization and concentration of NO and NO-derivates is critical for further advances in this area. Thirdly, considering that both *S*-nitrosylation and tyrosine nitration are apparently reversible events, more conclusive studies on the denitrosylation and denitration systems as well as the general turnover of *S*-nitrosylated and nitrated proteins in plant cells also seems a logical requirement for a deeper understanding of the dynamics of these regulatory processes. Similar to the action of protein phosphatases during the regulation of protein phosphorylation, denitrosylases and denitrases may possibly play an important role in defining the kinetics of the NO impacts on plant signaling cascades.

Another aspect that also deserves further attention is the potential existence of feed-forward cycles, in which NO modulates the production and/or signaling of specific plant hormones and these same hormonal species influence the machinery responsible for controlling NO endogenous levels. As described in the course of this review, accumulating pharmacological and genetic evidence demonstrates that representatives of virtually all classes of plant hormones may impact, at least at a certain degree, the endogenous concentration and/or distribution of NO and, also very frequently, literature data seems to indicate that changes in NO levels might trigger alterations on the metabolism and/or signaling of many, if not all, hormonal classes. It is not clear, however, whether these processes occur at the same place and time, which is critical for generating authentic feed-forward cycles involving these signaling substances. Naturally, a more complete characterization of the actual impacts of specific plant hormones on the NO biosynthetic and removal machinery (e.g., NR, NOA1, GSNOR, nsHbs) seems a key step in such research topic.

Additionally, as also discussed earlier in this review, NO might affect the signaling transduction of certain hormones, such as auxins and GAs, by modulating signaling elements (e.g., receptor, signaling transduction molecules) that impact the general dynamics of ubiquitination and proteasome-dependent degradation of repressor proteins (e.g., AUX/IAA and DELLA proteins). Interestingly, in animal systems, NO has consistently been shown to influence protein stability via regulation of ubiquitination and proteasome-dependent proteolysis (Hess et al., 2005) and, at least in humans, ubiquitin ligases themselves are targets of *S*-nitrosylation (Chung et al., 2004). Considering that several plant hormone signaling transduction mechanisms are based on the ubiquitination and subsequent proteasome-dependent degradation of repressor proteins, investigating whether NO might also directly affect this protein degradation labeling system in plants seems a promising venue for uncovering additional mechanisms possibly involved in NO signaling in plant systems.

Another intriguing question that remains to be answered is how plants can distinguish endogenously produced NO signals from the NO naturally present in the environment (e.g., atmosphere, rhizosphere). Whereas the gaseous and highly diffusible nature of NO may promote certain movement of this molecule inside the plant tissue and at the plant–environment interface, the high reactivity and inherent instability of NO may possibly limit the diffusion of this free radical through biological tissues. In several aspects, this seems a relevant and challenging question to be answered in the future.

Finally, we must remain open-minded to conceive increasingly complex NO–phytohormone interconnection nodes since new targets of NO-dependent PTMs and other upstream and downstream elements of NO signaling cascades will likely be identified in the future. At the same time, more complete pictures mechanistically explaining how multiple plant hormones may simultaneously interact with NO to control specific plant responses might also emerge, very likely leading to exciting new models of NO–phytohormone interaction networks. Moreover, this whole scenario will be further complicated when the intensive research conducted today in a restricted number of plant models (e.g., *Arabidopsis*, tomato, rice) is extended to a broader range of plant species and environmental contexts. Altogether, this knowledge will improve our ability to define the actual roles of NO during the regulation of the distinct plant responses controlled by this multipurpose signaling molecule and may also lead to new opportunities to manipulate NO–phytohormone interactions and, thus, regulate plant growth, development, and metabolism.

## Conflict of Interest Statement

The author declares that the research was conducted in the absence of any commercial or financial relationships that could be construed as a potential conflict of interest.
